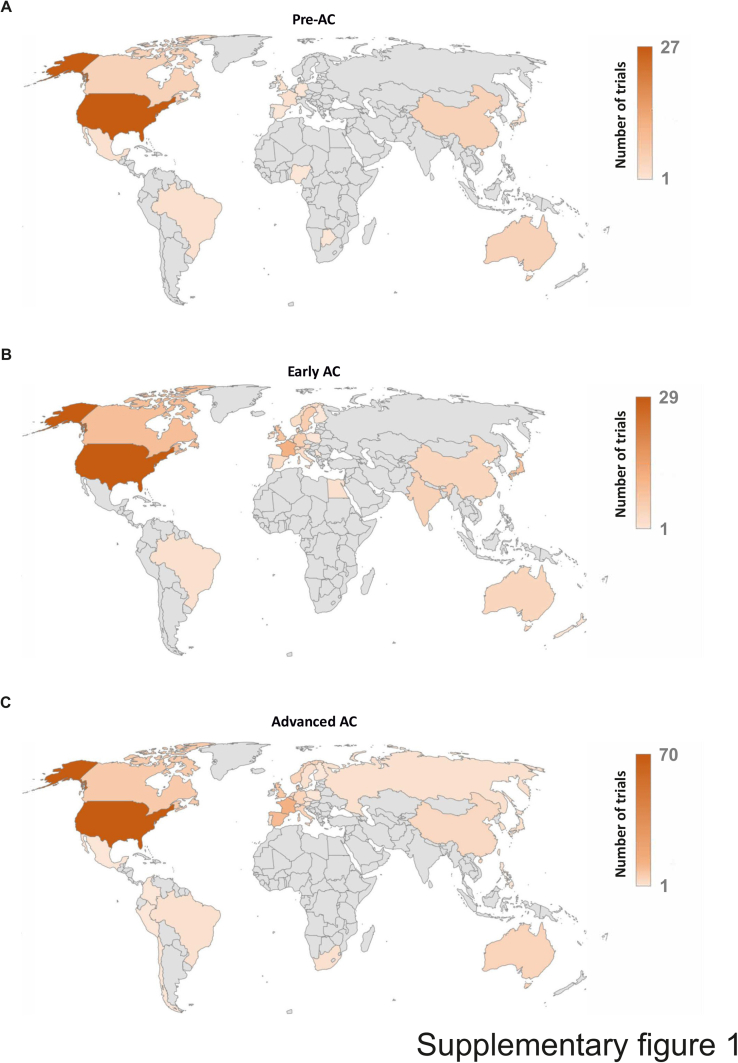# An overview of clinical trials in anal cancer 1999-2024

**DOI:** 10.1016/j.esmogo.2025.100145

**Published:** 2025-02-17

**Authors:** D. Verschoor, E.S.L. Pedersen, A. Tardit, E. Segelov

**Affiliations:** 1 Department of Clinical Research, University of Bern, Bern, Switzerland; 2 Department of Radiation Oncology, Inselspital, Bern University Hospital, University of Bern, Bern, Switzerland

**Keywords:** anal cancer, anal squamous-cell carcinoma, RCT, trials, international, HPV

## Abstract

**Background:**

Anal cancer (AC) is an uncommon malignancy. Clinical trials are crucial for improving prevention and treatment, but conducting research is challenging in this condition. This paper provides an overview of registered clinical trials in the prevention and treatment of AC between 1999 and 2024.

**Materials and methods:**

AC trials were identified from the two main global trial databases: the International Clinical Trials Registry Platform and clinicaltrials.gov. Trials were categorized based on context and disease extent: pre-AC (pre-cancerous states), early AC (localized, curable disease), and advanced AC (metastatic and/or incurable disease).

**Results:**

There were 251 trials identified: 55 pre-AC, 100 early AC, and 96 advanced AC. Only 10% were phase III studies. The number of trials increased over the 25-year period, particularly advanced AC trials since 2015. Trials listed as active totaled 109, of which 54% were actively recruiting. Most trials were conducted in high-income countries and only 38 were multinational. Underrepresented groups were immunosuppressed patients. Only one-third of trials were industry-sponsored, lower than most other cancer types.

**Conclusions:**

Although there has been a steady increase in AC trials, many research questions remain. Disparities are clear, with few trials in low-income countries, where the burden of disease is likely to grow as a consequence of less access to human papillomavirus (HPV) vaccination, which is likely to prevent disease. Clinical trials in AC rely mainly on academic consortia.

## Highlights

• AC is an uncommon form of HPV-related cancer, but the incidence is increasing.
• The clinical trial landscape of AC is informative for future studies.
• Future studies need to include low- and middle-income countries and disparities.
• Most trials are academic, with less industry sponsorship than for other cancers.

## Introduction

Anal cancer (AC) is an uncommon neoplasm affecting the mucosa-lined anal canal and less commonly the perianal skin.^1^ The most frequent histology is squamous-cell carcinoma (SCC), accounting for 75%-95% of cases, with the remainder adenocarcinoma (5%-15%) and very rare tumors including melanoma, neuroendocrine cancers, and metastases.^2^^,^^3^ Human papillomavirus (HPV) is estimated to cause up to 90% of anal SCC and the impact of HPV vaccination on prevention should be profound. AC usually presents as local or locoregional disease; metastatic disease is rare.^4^ AC incidence differs between population groups, being higher in females,^4^, ^5^, ^6^ older age, persons who have anal receptive intercourse,^7^, ^8^, ^9^ immunosuppression [especially people living with human immunodeficiency virus (HIV) and solid organ transplant recipients],^7^^,^^10^, ^11^, ^12^, ^13^ previous HPV-related cancers,^14^ and those with precursor lesions such as high-grade anal intraepithelial neoplasm (AIN).^7^^,^^9^^,^^13^^,^^15^^,^^16^ Anal SCC incidence has increased over the past decades,^2^^,^^17^ mainly in high-income countries (HIC), noting that little incidence data are available from low- and middle-income countries (LMIC).

Management of anal SCC has significantly changed since landmark clinical trials almost 40 years ago demonstrated that outcomes from concurrent chemoradiation were equivalent to the mutilating surgery of abdominoperineal resection, which removes fecal continence.^18^, ^19^, ^20^, ^21^ Although salvage surgery is required in up to 20% of patients due to persistent or recurrent local disease, most patients are still cured. Many trials are evaluating optimal methods for prediction of response to primary treatment. Management of metastatic disease has been limited to a small number of chemotherapy agents until recently when trials evaluating immune checkpoint inhibitors have reported efficacy.^20^^,^^22^

The prognosis for AC is good, with a 5-year overall survival of 75%.^23^, ^24^, ^25^ However, significant disparities remain, with poorer prognosis found in women, older age, people living with HIV, and Afro-American ethnicity.^26^^,^^27^

Clinical trials are essential drivers for improvement in prevention and treatment, yet the conduct of well-powered studies remains challenging.^28^ Much of the evidence for treatment effects relies on relatively small single-arm studies. Trials from LMIC are underrepresented. A global effort is needed to enroll patients particularly from poor prognostic groups or in settings where HPV vaccination uptake is low, as populations who do not receive primary HPV vaccination are a major concern for the future.

Quality-of-life outcomes are particularly important in AC, due to high survival rate and well-described physical, sexual, and psychological impact of the disease and its treatment. Optimal screening for premalignant lesions is also the subject of much research, to delineate steps in the evolution from low- to high-grade AIN, which can regress or progress to cancer, and to understand how HPV subtype affects prognosis, as per cervical cancer.^29^ Furthermore, screening gives the opportunity for prevention, as early identification and management of premalignant lesions can interrupt the progression to invasive disease.^30^

This article provides a comprehensive overview of the past 25 years of clinical trials in AC, to inform gaps and understand the global research effort across the prevention–treatment spectrum.

## Materials and methods

### Data collection

AC trials were identified from the two main global trial databases: the International Clinical Trials Registry Platform (ICTRP; a World Health Organization platform comprising data from 20 registries^31^) and the clinicaltrials.gov platform.^32^ A detailed search query containing 64 terms as synonyms of AC was used to collect data from trials registered as of 5 February 2024 (Supplementary Material and Methods, available at https://doi.org/10.1016/j.esmogo.2025.100145). Duplicates and observational studies were excluded. Primary outcomes, HIV status, HPV status, and strain were analyzed and categorized, and verified by a second researcher.

### Data preparation

Trials were categorized into three groups, based on the most advanced disease extent included:

- Pre-AC: includes people at high risk for developing AC but with no prior or current AC diagnosis; with either HIV positivity, prior HPV-related malignancy, or *in situ* anal neoplasms.
- Early AC: AC stage T1-4, N0-1, M0 using Union for International Cancer Control Tumor Node Metastasis (UICC TNM eighth edition).^20^
- Advanced AC: metastatic, recurrent, and incurable and/or refractory AC.

### Statistical analysis

Characteristics were described for all trials and by subgroup (pre-AC, early AC, and advanced AC) using number with percentage and medians with full or interquartile range. Stata version 18 (StataCorp LLC, College Station, TX) was used for analyses.

## Results

### Clinical trial landscape

**Figure 1:**
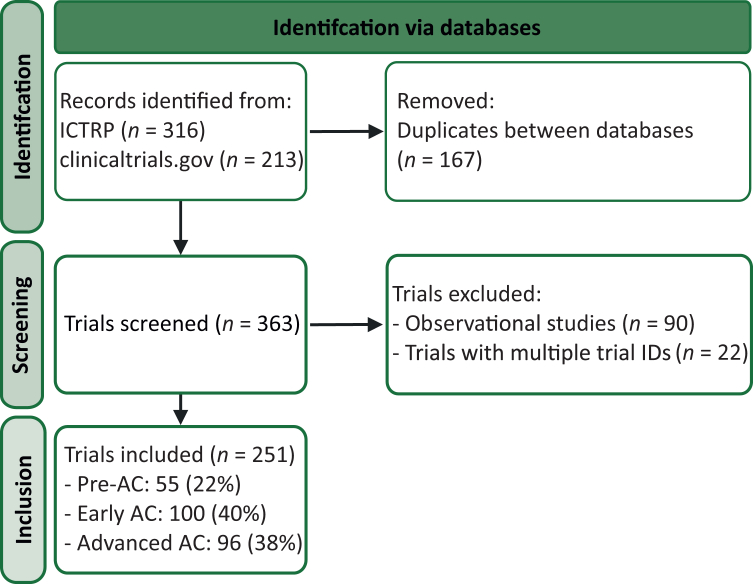
CONSORT diagram. ICTRP, International Clinical Trials Registry Platform.

A total of 251 trials met inclusion criteria, from 316 trials in ICTRP and 213 in clinicaltrials.gov (Figure 1). Of these, 55 were in pre-AC (22%), 100 in early AC (40%), and 96 in advanced AC (38%).

**Figure 2:**
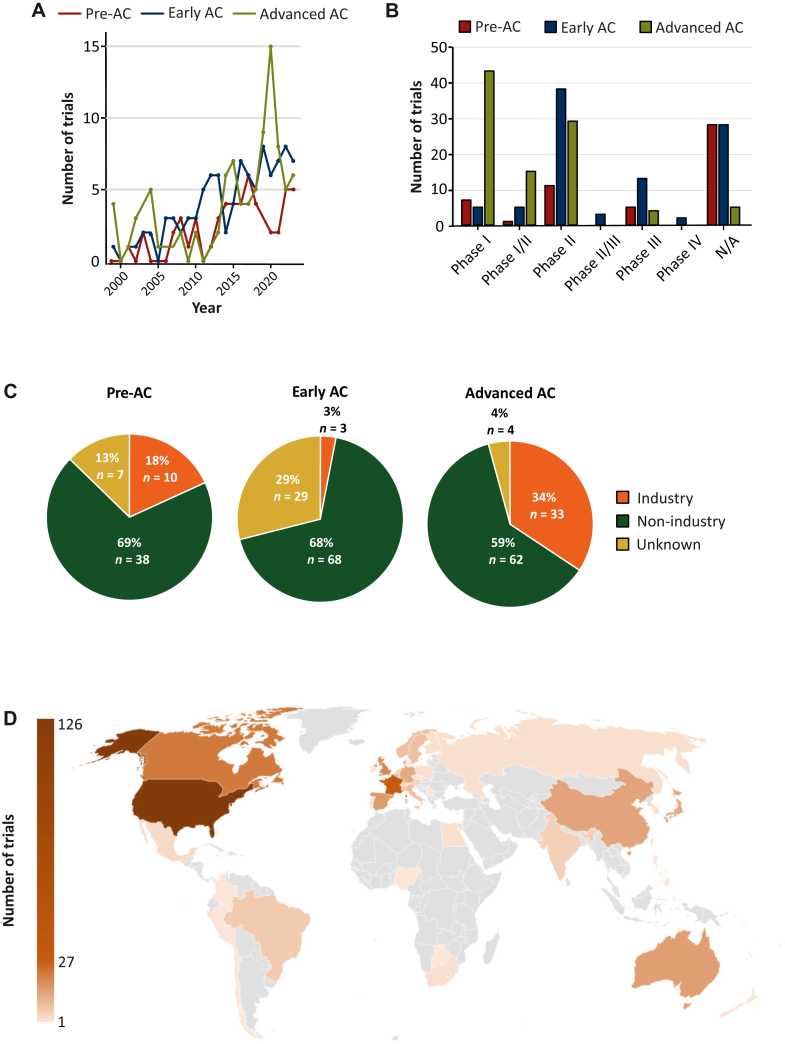
**Overview of anal cancer clinical trial registrations.** (A) Number of new anal cancer trial registrations per year (two trials in early AC registered in 2024 are not represented). (B) Trial registrations by phase. (C) Trial sponsorship. (D) Map showing countries where trials were conducted. AC, anal cancer.

The number of trials steadily increased to an average of six per year between 2015 and 2023, with a notable recent increase in trials for advanced disease to a peak of 15 trials in 2020 (Figure 2A). Most trials were phase I or II (Figure 2B); only 25 (10%) were phase III trials. In pre-AC and early AC, phase was commonly listed as non-applicable; these trials mainly evaluated devices or behavioral interventions. Industry rarely sponsored preventative or early disease trials; even for advanced disease, only one-third of trials listed industry as sponsor (Figure 2C). Approximately 40% of trials were each completed or active, with the remainder terminated (6%), withdrawn (2%), or unknown (8%) (Table 1). Of the 109 active trials, 59 (54%) were currently recruiting.

**Table 1:** Characteristics of registered trials in anal cancer 1999-2024

	Total	Pre-AC	Early AC	Advanced AC
	*n* = 251	*n* = 55	*n* = 100	*n* = 96
	*n* (%)	*n* (%)	*n* (%)	*n* (%)
Status				
Active	109 (43)	18 (33)	44 (44)	47 (49)
Not yet recruiting	12 (11)	2 (11)	8 (18)	2 (4)
Recruiting	59 (54)	9 (50)	22 (50)	28 (60)
Active, not recruiting	38 (35)	7 (39)	14 (32)	17 (34)
Completed	100 (40)	27 (49)	34 (34)	39 (41)
Terminated	16 (6)	2 (4)	11 (11)	3 (3)
Withdrawn	5 (2)	1 (2)	1 (1)	3 (3)
Unknown status	21 (8)	7 (13)	10 (10)	4 (4)
Multinational (*n* = 241)^a^				
Yes	38 (16)	7 (13)	12 (13)	19 (16)
*n* countries median (range)	2 (2-23)	2 (2-2)	3 (2-11)	3 (2-23)
Purpose				
Treatment	173 (69)	16 (29)	69 (69)	88 (92)
Diagnostic	9 (4)	2 (4)	7 /7)	-
Prevention	27 (11)	23 (42)	4 (4)	-
Screening	10 (4)	10 (18)	-	-
Safety	5 (2)	-	2 (2)	3 (3)
Supportive care	11 (4)	-	9 (9)	2 (2)
Other^b^	16 (6)	4 (7)	9 (9)	3 (3)
Primary outcomes				
Adverse effects and complications	63 (25)	7 (13)	27 (27)	29 (30)
Response related	62 (25)	8 (15)	24 (24)	30 (31)
Dose related	50 (20)	3 (5)	11 (11)	36 (38)
Survival related	39 (16)	2 (4)	22 (22)	15 (16)
Treatment and intervention	15 (6)	6 (11)	7 (7)	2 (2)
Quality of life, pain, and questionnaires	13 (5)	2 (4)	10 (10)	1 (1)
Feasibility and efficacy	12 (5)	1 (1)	6 (6)	5 (5)
Diagnosis and incidence	12 (5)	11 (20)	1 (1)	-
Therapy performance	11 (4)	2 (4)	8 (8)	1 (1)
Detection sensitivity and specificity	10 (4)	10 (18)	-	-
Immunological response	10 (4)	4 (7)	-	6 (6)
Patients and managerial	9 (4)	3 (5)	4 (4)	2 (2)
Infection and vaccination	5 (2)	2 (4)	3 (3)	-
Other^c^	19 (8)	4 (7)	12 (12)	3 (3)
Unknown	17 (7)	4 (7)	3 (3)	10 (10)

Data extracted from ICTRP and clinicaltrials.gov databases on 5 February 2024. Where trials listed multiple primary outcomes, each was recorded. Data are presented as entered and not recategorized.

AC, anal cancer; ICTRP, International Clinical Trials Registry Platform.

a Ten trials did not have information available.

b Includes health service research = 3, change in care = 1, unknown = 12.

c Includes pathological and clinical status = 4, gastrointestinal symptom and physiological function = 3, pharmacokinetics = 3, laboratory = 2, vital signs = 3, duration related = 2, sexual function = 1, feasibility = 1.

### Design characteristics

Trials were conducted across the globe; however, most were located in HIC and the spread by group was similar (Figure 2D, Supplementary Figure S1, available at https://doi.org/10.1016/j.esmogo.2025.100145). Few trials were conducted in the African, Middle Eastern, or Asian regions. Only 16% of trials were multinational (*n* = 38), with a median number of countries of only 2 (range 2-23). In pre-AC, prevention was the most common purpose (42%), with primary outcome related to diagnosis or disease detection sensitivity (Table 1). As expected, in advanced AC, treatment was the dominant purpose (92%), with primary outcomes related to response and/or adverse events.

**Figure 3:**
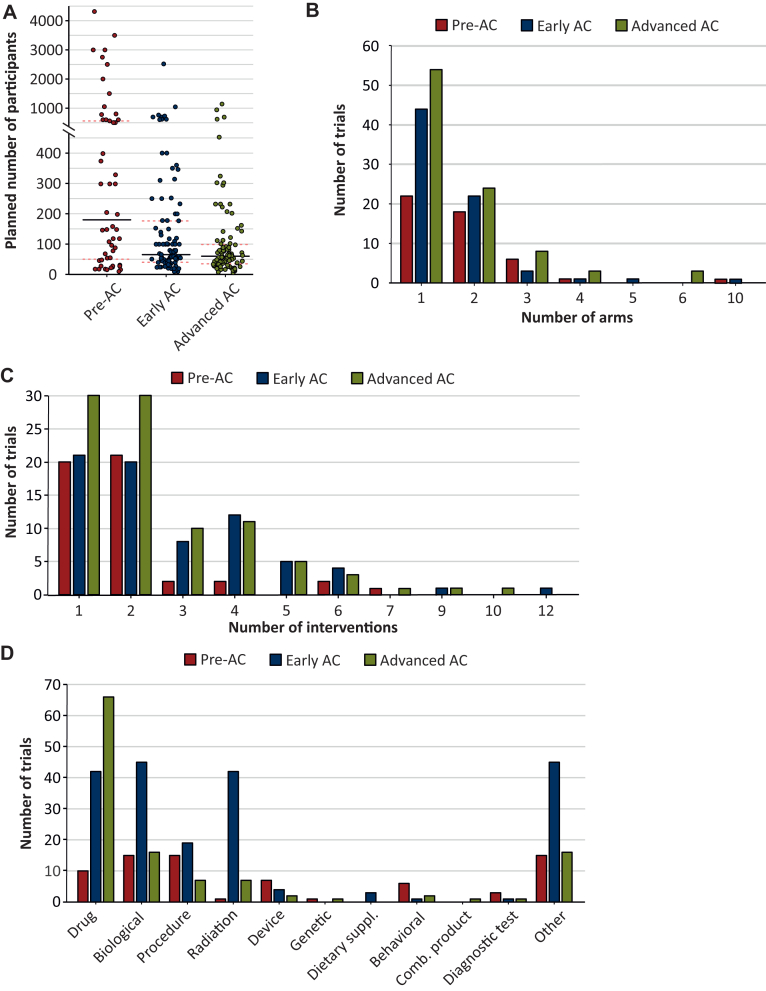
**Design characteristics of anal cancer trials registered in clinicaltrials.gov**. (A) Recruitment targets. Solid line is median, broken lines mark quartiles 1 and 3. (B) Number of study arms. (C) Number of interventions (D) Type of intervention. comb product, combination product; dietary suppl, dietary supplement.

Over the 25-year period, trials aimed to recruit a total of 65 826 participants: 38 317 for pre-AC, 16 975 for early AC, and 10 534 for advanced AC (Figure 3A). The median target recruitment for pre-AC trials was 180 (range 12-5058) people, for early AC was 66 (range 10-2500) people, and advanced AC was 60 (range 4-1100) people.

Trials listed in clinicaltrials.gov were able to be analyzed for number of arms and number and type of interventions. Most trials had one or two arms (87%; Figure 3B); similarly most trials had either one or two interventions (67%; Figure 3C). Of the interventions, 47% were classified as drug, 21% as biological, 20% as radiation, and 16% as procedure (Figure 3D).

### Eligibility criteria

Trials studied adult populations and most included both males and females, with some difference between groups: pre-AC trials included either only females (16%) or males (31%) while advanced AC trials included both (99%) (Table 2). Inclusion of people living with HIV or according to HPV status also varied. Exclusive inclusion of people living with HIV was only common in pre-AC trials (36%), compared with 2% in early and advanced trials. Exclusion of people living with HIV was mostly observed in advanced AC trials (35%). Only 12% of trials stated used HPV positivity as an inclusion criterion. Of these, almost all included HPV16 exclusively (22 of 31, 71%); only three trials included HPV16 and 18, and two trials exclusively included HPV18.

**Table 2:** Inclusion criteria of registered trials in anal cancer 1999-2024

	Total	Pre-AC	Early AC	Advanced AC
	*n* = 251	*n* = 55	*n* = 100	*n* = 96
	*n* (%)	*n* (%)	*n* (%)	*n* (%)
Lower age limit				
<18 years	18 (7)	8 (15)	9 (9)	1 (1)
18-29 years	223 (93)	47 (85)	91 (91)	95 (99)
Sex				
Females and males	221 (88)	29 (53)	97 (97)	95 (99)
Females only	13 (5)	9 (16)	3 (3)	1 (1)
Males only	17 (7)	17 (31)	—	—
People living with HIV				
Exclusively included	24 (10)	20 (36)	2 (2)	2 (2)
Included	56 (22)	12 (22)	20 (20)	24 (25)
Excluded	60 (24)	10 (18)	16 (16)	34 (35)
Not specified	111 (44)	13 (24)	62 (62)	36 (38)
HPV status				
HPV positive	31 (12)	5 (9)	3 (3)	23 (24)
HPV 16	22 (71)	1 (20)	3 (100)	18 (78)
HPV 16 and 18	3 (10)	1 (20)	—	2 (9)
HPV 18	2 (6)	—	—	2 (9)
Not specified	4 (13)	3 (60)	—	1 (4)
HPV negative	3 (1)	2 (4)	1 (1)	—
Not specified	217 (86)	48 (87)	96 (96)	73 (76)

Data extracted from ICTRP and clinicaltrials.gov databases on 5 February 2024.

AC, anal cancer; HIV, human immunodeficiency virus; HPV, human papillomavirus; ICTRP, International Clinical Trials Registry Platform.

## Discussion

This comprehensive analysis of existing and planned clinical trials in AC over the past 25 years allows an appreciation of trends, gaps, and disparities that should be thoughtfully and systemically addressed by the global, academic consortia who are most likely to undertake research in this complex disease. It is striking that unlike many other cancers, large phase III randomized trials are infrequent, and industry sponsorship is uncommon.^33^ Even for advanced disease, due to its rarity, commercial sponsorship is difficult to secure, leaving trials with limited funding and restricting capacity for added value functionality such as biospecimen collection, due to high costs.

Our results showed that the majority of clinical trials are conducted in high-income regions, with few to no trials in areas with high HPV and HIV infection. Health care settings and population risk factors are often so different in low-income countries, that findings from high-income settings may not be transferrable.^34^ Further, while incidence of AC is currently highest in HIC,^5^^,^^17^ this is likely to shift toward AC predominating in LMIC, as the preventative benefit of HPV vaccination may be delayed, due to lower vaccination coverage and/or uptake, and lack of offering of vaccinations to males.^35^^,^^36^ Less reliable data collection, which particularly affects rarer cancers, compounds the problem due to lack of national registries.^37^ Proactive steps to manage this emerging problem should be taken, including efforts to include LMIC in trials. Streamlined and pragmatic trials appropriate for such health systems should be encouraged and funded by global health bodies.

In the era of recognition of gender medicine as an important determiner of outcome for various diseases, the inferior outcome of AC in women, as well the other poor prognostic groups, should also be addressed purposefully in trial design.^38^ Identifying higher risk groups and tailoring interventions are particularly difficult in rarer diseases, but newer trial designs using Bayesian methodologies are better equipped to accommodate such complexities.

Study strengths include analysis of the two largest clinical trial databases worldwide, which comprehensively cover the vast majority of trials.^39^ Limitations are that the accuracy of the presented data relies on the quality of information entered in the clinical trial databases. However, both databases have quality management processes involving review of uploaded data.

### Conclusion

Trials addressing key questions across the spectrum of AC have increased steadily over the past two decades, but more is needed to address the many outstanding research questions in this uncommon cancer. Global academic trial consortia need adequate infrastructure and funding support to locate trials to reflect current and emerging global disease burden.

## Figures and Tables

**Supplementary Figure 1:**