# Supplementary material for: An overview of clinical trials in anal cancer 1999-2024
Source: ESMO Gastrointest Oncol. 2025 Feb 17;13(Pt A):100145. doi: 10.1016/j.esmogo.2025.100145 (PMC13459589; doi:10.1016/j.esmogo.2025.100145)
Supplement: Supplementary file 1 [file mmc1.docx]

**Appendices:**

Supplementary Material & Methods:

The search query:

"adenocarcinoma of anal canal" OR "adenocarcinoma of anus" OR "adenocarcinoma of the anal canal" OR "adenocarcinoma of the anus" OR "anal adenocarcinoma" OR "anal canal adenocarcinoma" OR "anal canal cancer" OR "anal canal carcinoma" OR "anal canal neoplasm" OR "anal canal tumor" OR "anal cancer" OR "anal carcinoma" OR "anal neoplasm" OR "anal tumor" OR "anus adenocarcinoma" OR "anus cancer" OR "anus carcinoma" OR "anus neoplasm" OR "anus tumor" OR "cancer of anal canal" OR "cancer of anus" OR "cancer of the anal canal" OR "cancer of the anus" OR "carcinoma of anal canal" OR "carcinoma of anus" OR "carcinoma of the anal canal" OR "carcinoma of the anus" OR "neoplasm of anal canal" OR "neoplasm of anus" OR "neoplasm of the anal canal" OR "neoplasm of the anus" OR "scc of anal canal" OR "scc of anus" OR "scc of the anal canal" OR "scc of the anus" OR "squamous cell anal neoplasm" OR "squamous cell adenocarcinoma of anal canal" OR "squamous cell adenocarcinoma of anus" OR "squamous cell adenocarcinoma of the anal canal" OR "squamous cell adenocarcinoma of the anus" OR "squamous cell anal adenocarcinoma" OR "squamous cell anal cancer" OR "squamous cell anal carcinoma" OR "squamous cell anal tumor" OR "squamous cell cancer of anal canal" OR "squamous cell cancer of anus" OR "squamous cell cancer of the anal canal" OR "squamous cell cancer of the anus" OR "squamous cell carcinoma of anal canal" OR "squamous cell carcinoma of anus" OR "squamous cell carcinoma of the anal canal" OR "squamous cell carcinoma of the anus" OR "squamous cell neoplasm of anal canal" OR "squamous cell neoplasm of anus" OR "squamous cell neoplasm of the anal canal" OR "squamous cell neoplasm of the anus" OR "squamous cell tumor of anal canal" OR "squamous cell tumor of anus" OR "squamous cell tumor of the anal canal" OR "squamous cell tumor of the anus" OR "tumor of anal canal" OR "tumor of anus" OR "tumor of the anal canal" OR "tumor of the anus"

Data collected:

TrialID, Public title, Scientific title, Acronym/other ID, web address, Date registration, Overall status, Termination reason, Phase, Primary Purpose, Target size, Location (Countries), Intervention, Primary outcome, Primary sponsor, Sponsor type, Age, Gender, Inclusion Criteria, Exclusion Criteria, Condition
